# Accommodating Serial Correlation and Sequential Design Elements in Personalized Studies and Aggregated Personalized Studies

**DOI:** 10.1162/99608f92.f1eef6f4

**Published:** 2022-09-08

**Authors:** Nicholas J. Schork

**Affiliations:** The Translational Genomics Research Institute (TGen), an affiliate of the City of Hope National Medical Center; The University of California San Diego; and Scripps Research

**Keywords:** precision medicine, serial correlation, sequential analysis, drug development

## Abstract

Single subject, or ‘N-of-1,’ studies are receiving a great deal of attention from both theoretical and applied researchers. This is consistent with the growing acceptance of ‘personalized’ approaches to health care and the need to prove that personalized interventions tailored to an individual’s likely unique physiological profile and other characteristics work as they should. In fact, the preferred way of referring to N-of-1 studies in contemporary settings is as ‘personalized studies.’ Designing efficient personalized studies and analyzing data from them in ways that ensure statistically valid inferences are not trivial, however. I briefly discuss some of the more complex issues surrounding the design and analysis of personalized studies, such as the use of washout periods, the frequency with which measures associated with the efficacy of an intervention are collected during a study, and the serious effect that serial correlation can have on the analysis and interpretation of personalized study data and results if not accounted for explicitly. I point out that more efficient sequential designs for personalized and aggregated personalized studies can be developed, and I explore the properties of sequential personalized studies in a few settings via simulation studies. Finally, I comment on contexts within which personalized studies will likely be pursued in the future.

## Media Summary

The introduction of new therapies or disease prevention interventions must come with a commitment to determine if they actually provide benefit. To achieve this, relevant studies typically focus on the effects that a therapy or intervention might have on a randomly chosen individual with certain characteristics (like a disease or frequently observed disease-risk profile). As a result, such studies often provide insight into the average or population-level effects that the therapy or intervention has, addressing questions such as: ‘Do many people benefit based on some predefined criteria of benefit?’ ‘How many people exhibit side effects?’ and ‘Does the new therapy or intervention benefit more people than another therapy or intervention?’ Such studies rarely collect enough information on any one individual to unequivocally characterize the nature of that individual’s response and therefore do not address questions such as: ‘Does the therapy affect that individual to a quantifiably greater or lesser degree than other individuals?’ ‘Are there factors unique to that individual, for example, aspects of their diet or other medications they are on, that affect their response at any given time?’ and ‘Does the therapy affect other aspects of the health of that individual, for example, their sleep, in deterministic ways?’ Studies designed to explore individual responses to a therapy or intervention are referred to, unsurprisingly, as single subject, ‘N-of-1’ or, preferentially, as ‘personalized’ studies. Personalized studies can be complicated and expensive to pursue, especially if a goal is to aggregate the results of many personalized studies to, for example, determine how many people exhibit similar overall responses. In addition, personalized studies must be designed and analyzed in ways that are sensitive to the use of washout periods, serial correlation among the measurements made during the study, and other phenomena. I describe some of the challenges in the design and conduct of personalized studies and propose making them more efficient in certain contexts by analyzing the data collected during their execution sequentially; that is, making decisions about the effects of an intervention on an individual in real time, stopping the study at any point in which sufficient data have been collected to make compelling claims about the efficacy (or lack thereof) of the intervention. I use simulation studies to explore the properties of personalized studies, including sequential personalized studies. I also briefly mention a few future directions for N-of-1 studies that build off the proposed study designs and issues discussed.

## 1. Introduction: Traditional vs. N-of-1 Studies of Interventions

### 1.1. Population-Based Randomized Controlled Trials (RCTs)

Most researchers developing interventions, whether therapeutic, palliative, or preventive, want to know if their interventions benefit people and have the effects that they were designed to have. Since a typical motivation to develop an intervention is a need in the population at large, and many people might benefit from the intervention as a result, most studies focus on the benefits of the intervention in the population at large. Relevant studies might focus on the average effect of an intervention over a large number of people, seeking to show that it is likely to positively benefit more people than, say, a comparator intervention, which could be a placebo. The design of such studies has received a great deal of attention over the years, with emphasis on variations of traditional population-based randomized controlled trials, or RCTs (Friedman, Furberg, and DeMets 2010)(Meeker-O’Connell et al. 2016)(Pawlik and Sosa 2020). Traditional population-based RCTs can be quite complex, but the basic strategy behind them involves assigning some number of enrollees in a trial the intervention whose efficacy is in question, while others are assigned to a placebo or comparator intervention. The effects of the drug are measured in both groups and compared to determine the merits of the experimental intervention relative to the comparator intervention. The assignments as to which enrollees in the trial receive the experimental intervention or the comparator are done randomly so that those receiving the experimental intervention have a high probability of having the same characteristics as those receiving the comparator intervention to ensure that any observed effect of the experimental intervention is likely to be causal (Collins et al. 2020)(Deaton and Cartwright 2018).

RCTs are often required, justifiably, by regulatory agencies to make sure new interventions have the positive benefit they are claimed to have. I will not cover all the challenges and methods used in the conduct of traditional RCTs, but suffice it to say that there is a rather voluminous literature on the subject, with some of it questioning the fundamental tenets behind population-based RCTs, such as the belief that randomization in the assignment of experimental and comparator interventions to enrollees in a trial can achieve what it is intended to do, or the belief that RCTs could be supplanted by other study designs (Deaton and Cartwright 2018)(Nicholas J. Schork 2018)(Subramanian, Kim, and Christakis 2018). An important and very consequential point of emphasis about traditional population-based RCTs is that it is an open question as to what constitutes sufficient population-level benefit to motivate the use of an intervention based on the results of a population-based RCT, as many interventions have been unequivocally shown *not* to work on all the individuals who take them based on a variety of different population-based metrics exploring intervention responses (Nicholas J. Schork 2015)(Senn 2018)(Subramanian, Kim, and Christakis 2018). This suggests that either standard population RCTs and metrics used to assess the utility of an intervention in the population at large are flawed, or the biomedical science community needs to broadly rethink current health care practices and ways in which new interventions and health care technologies are vetted and adopted.

### 1.2. The Emergence of Precision Medicine

The fact that interventions do not work for everyone raises the question as to why. There is overwhelming evidence that the often very nuanced or even unique characteristics that an individual possesses, such as their genetic, physiologic, dietary, and behavioral profile, as well their history of exposures to various substances and access to health care, can all influence their response to an intervention. In fact, the evidence is so pronounced that very large-scale efforts promoting ‘precision,’ ‘individualized,’ or ‘personalized’ approaches to health care—in which individual characteristics are used to tailor interventions to an individual—have been promoted and even adopted in contemporary medical and public health text books (n.d.a)(n.d.b)(n.d.c)(n.d.d). Proving that a particular individual responds to an intervention, or that the individual’s response to an intervention is shaped by very nuanced features they have is not trivial, and requires study designs that go beyond and complement those adopted in traditional population-based RCTs. There is a growing literature on single subject, ‘N-of-1,’ or what are now more preferentially referred to as ‘personalized’ study designs that are meant to probe individual response to interventions (Chapple and Blackston 2019)(n.d.e)(Kravitz, Schmid, and Sim 2019)(Lillie et al. 2011)(McDonald, McGree, and Bazzano 2019)(Nicholas J. Schork and Goetz 2017). The use of the term ‘N-of-1’ reflects the fact that the sample size in terms of the number of units of observation or individuals in such studies is 1. However, the term ‘personalized’ in such contexts is more intuitive for many. Personalized studies can be pursued for a variety of reasons, however. For example, it may be the case that the condition for which an intervention has been designed is very rare (N. J. Schork and Nazor 2017) and hence must be tailored to profiles of the few people with the condition, or, as discussed later, the interventions being tested are truly personalized, like CAR-T cells for cancer, and hence not likely to work in anyone but the specific individual they were designed for (Nicholas J. Schork et al. 2020). As a last example, there may be a need to focus on very detailed evaluations of an individual receiving an intervention because the intervention has a small therapeutic window (i.e., range of dosages for which a positive effect is expected and for which no side effects are likely to occur) and thus requires a very careful administration and monitoring of its affects (Cremers, Guha, and Shine 2016). Note that methods for exploring population-level heterogeneity in patient responses to interventions based on data generated from general or traditional RCTs have been explored by many researchers, and the results of these studies have revealed evidence motivating personalized medicine strategies in many instances (n.d.f)(Schandelmaier et al. 2020).

### 1.3. Aggregating Personalized Studies and Choosing a Design for Their Execution

The results of personalized studies can be aggregated to explore more population-level phenomena, like the fraction of individuals likely to have unique responses based on characteristics they have (Blackston et al. 2019)(Punja et al. 2016). Note that in this context it is very important to define some measure of the clinical impact of an intervention, in terms of an effect size, and use this to determine what fraction of individuals might be responding to the intervention, or very unfortunate and erroneous claims about biological variability and the clinical utility of the intervention could be made. Consider, for example, a situation in which individuals vary appreciably in their response to an intervention to treat a disease they all have, but none of their responses is large enough to actually impact their disease course in meaningful ways. In this situation, biologically meaningful variability may exist in the responses to intervention, but this variation will not lead to the identification of individuals who will ultimately benefit from the intervention. In the following, I will not address what may be a clinically meaningful effect size, but rather discuss the basic design of personalized studies and some statistical challenges they face. It is also important to point out that, depending on the context, personalized studies can be expensive and logistically challenging (e.g., using a sophisticated continuous monitoring device on an individual to record their response to an intervention over the course of a lengthy study). In this light, one could ask, about a particular intervention, if there are enough resources to collect 1,000 response measurements through a device capable of use in the field (e.g., a portable blood pressure measurement monitoring device) to explore the benefits of an emerging intervention for hypertension; would it be best to get 1 measurement on 500 individuals provided the intervention and 1 measurement on 500 individuals provided a comparator intervention, or 500 measurements on a single individual while provided the intervention, and 500 measurements on that same individual while provided a comparator intervention? Obviously, it depends on the question, but this is an important trade-off in terms of resource utilization for making population-level benefit claims vs. individual benefit claims about an intervention. This trade-off can be complicated, as there are unique challenges in designing and executing both large-scale traditional RCTS and personalized trials, although there are many emerging and cost-effective health monitoring technologies that can lead to efficient and cost-effective population-level RCTs, as well as personalized studies, in different contexts (Anderson et al. 2020)(Cha et al. 2019)(Chung, Fortunato, and Radacsi 2019)(Herrington, Goldsack, and Landray 2018)(Marra et al. 2020)(n.d.g)(Topol, Steinhubl, and Torkamani 2015).

## 2. Challenges in the Design and Analysis of N-of-1 Trials

### 2.1. The Basic N-of-1 Design and Its Challenges

Most N-of-1 studies exploit some kind of variant of a basic crossover design (Lillie et al. 2011)(Wang and Schork 2019). Basically, an individual is provided a particular intervention (call it ‘intervention A’) and measures of their response to that intervention are recorded. The individual is then provided a comparator or placebo intervention (‘intervention B’) and measures of their response are also recorded. The response measures collected during the administration of each intervention are compared to make claims about the relative benefits of interventions A and B. Many phenomena can impact the power of a personalized study; for example, the length of time an individual is provided each intervention given, for example, the time it takes for the initiation of the activity of the intervention and its half-life in the body; the number of response measurements collected during each intervention period; the number of periods in which an individual is provided intervention A or B; the use of randomization to determine the order in which the interventions are provided; the use of initial baseline evaluation and washout periods (i.e., times when the individual is taken off an intervention to remove any lingering, or ‘carryover,’ effects of the intervention—note that the use of washouts can be controversial if an individual is being treated for a life-threatening condition and cannot afford to be off an intervention); whether response data are collected during washout periods and these data are considered in the analysis; the nature of the comparator intervention or interventions being studied (i.e., there is no reason multiple interventions cannot be evaluated); the collection and analysis of covariables (e.g., diet and activity) during the study to reduce confounding of intervention/response relationships; and the use of multiple response variables. These are all important considerations and impact the practicality, statistical power, and overall rigor of the study. Note also that since measures are made on an individual, there may be a time or learning effect when the measures are collected that masks as an intervention effect. For example, if an intervention is being explored to enhance cognitive ability as measured through an online reaction time or memory test, then the individual in the trial may simply get better at the task over time. Time and learning effects can be accommodated and controlled for, however, to some degree, through the use of covariates in the analysis model that reflect the times at which the measures have been made. There are a number of resources that can be used to design personalized trials. For example, Dudley and colleagues have developed smartphone apps for executing personalized trials (Badgeley et al. 2016)(Percha et al. 2019); (Senn 2019) has considered the sample sizes necessary to test certain hypotheses in personalized trials; and (n.d.h) provide a comprehensive review of general design and analytic considerations for personalized trials. In addition, there are a growing number of internet resources devoted to personalized and N-of-1 trials, for example, the website maintained by the International Collaborative Network for N-of-1 Clinical Trials and Single-Case Designs (Nikles et al. 2021).

### 2.2. Adaptive Designs vs. N-of-1 Trials

Adaptive or dynamic study designs for characterizing and optimizing the choice of an intervention for an individual are receiving a great deal of attention since they share some motivation and design concepts associated with traditional population-based RCTs and personalized studies (Kosorok and Moodie 2015)(Tsiatis et al. 2019). These designs consider, for example, randomizing individuals to one of a set of interventions of interest initially and then, as the data accrue on the individuals’ responses to those interventions, each individual is steered toward the intervention that the data suggest should work best for them. It is not without reason that these designs have elements in them of study designs that have been referred to as ‘play the winner’ designs (Rosenberger 1999). Adaptive designs can lead to dynamic treatment regimes (DTRs) in which interventions (‘treatments’) are potentially changed on the basis of information collected on an individual patient. Adaptive designs should continue to receive attention since it is complicated ethically to keep providing an individual an intervention that may not be benefitting them simply to ensure that statistical power for testing an intervention’s efficacy or inefficacy can be obtained (Cheung, Chakraborty, and Davidson 2015). In this light, minimizing the amount of time an individual is receiving what is likely to turn out to be an inferior intervention relative to others is appropriate ethically. Adaptive designs therefore have a place in biomedical research, but are not necessarily a substitute for personalized or N-of-1 studies, or even aggregated personalized studies, which have a dual focus on ‘within’ and ‘between’ individual variation in intervention response. Personalized studies, as noted, should therefore be designed to have enough statistical power to detect factors influencing within-individual variation in response to the intervention. Adaptive designs exploit population variation in a way that could inform studies of factors responsible for within-individual variation, but focus on between-individual variation in response to a set of interventions.

### 2.3. The Effects of Serial Correlation

Since N-of-1 studies focus on measurements obtained on a single individual over time while the individual is provided different interventions, the measurements are likely to exhibit serial (or auto-) correlation. Accommodating, measuring, exploiting, and avoiding serial correlation in time-series data are the subjects of a great deal of research among statisticians and data analysts, so I will not go into detail about the topic here (n.d.i), but rather focus on the consequences of serial correlation in personalized studies. An excellent source on the subject is provided in a book chapter by (n.d.j); although others have introduced the subject as well (n.d.k)(n.d.l)(Tang and Landes 2020)(Wang and Schork 2019). Serial correlation will likely arise if the response measures are collected with very short time intervals between them. For example, if a continuous monitor, say for electrodermal activity (EDA) stress response evaluation is used, then time-adjacent measures are likely to exhibit strong correlations. If the response measures are obtained with longer between-measurement time intervals, for example, once a week, they are not likely to exhibit as strong a serial correlation. In addition, the use of washout periods can influence the impact that serial correlation will have on type I and type II statistical error rates in drawing inferences about an intervention effect. This was pointed out by (Wang and Schork 2019) but, unfortunately, they did not consider the full range of phenomena that might be at play in evaluating the effects of serial correlation on personalized studies with and without washout periods. In addition, there are many forms of serial correlation that could affect a personalized trial. (Wang and Schork 2019) and (Rochon 1990) considered simple autoregressive order 1 (AR-1) models of serial correlation, which I also consider here, but clearly more work in this area should be pursued.

To expose the issues with serial correlation that can be exacerbated with the use of washout periods, consider the fact that serial correlation in a random variate in a time series not subjected to any perturbations can create ‘local’ mean differences in that variate during reasonably long segments of time, especially if strong serial correlation is present. Thus, two long stretches of time that are not adjacent may exhibit average value differences in the variate purely by chance if not accounted for properly. The probability that this will occur if the two stretches of time are adjacent (e.g., at time points 1–100 and 101–200 instead of, e.g., at time points 1–100 and 201–300) is lower since the ‘carryover’ of the correlations from the first segment to the adjacent second segment will reduce the chance of average differences between those two segments. Thus, if during a personalized study the outcome variable exhibits significant serial correlation, then the response observations obtained while the individual is provided, for example, intervention A may exhibit an average of that response variable that is different than the average of the response variable while the individual is provided intervention B purely by chance, if those periods are separated by a washout period and the serial correlation is not accounted for statistically.

**Figure 1.**
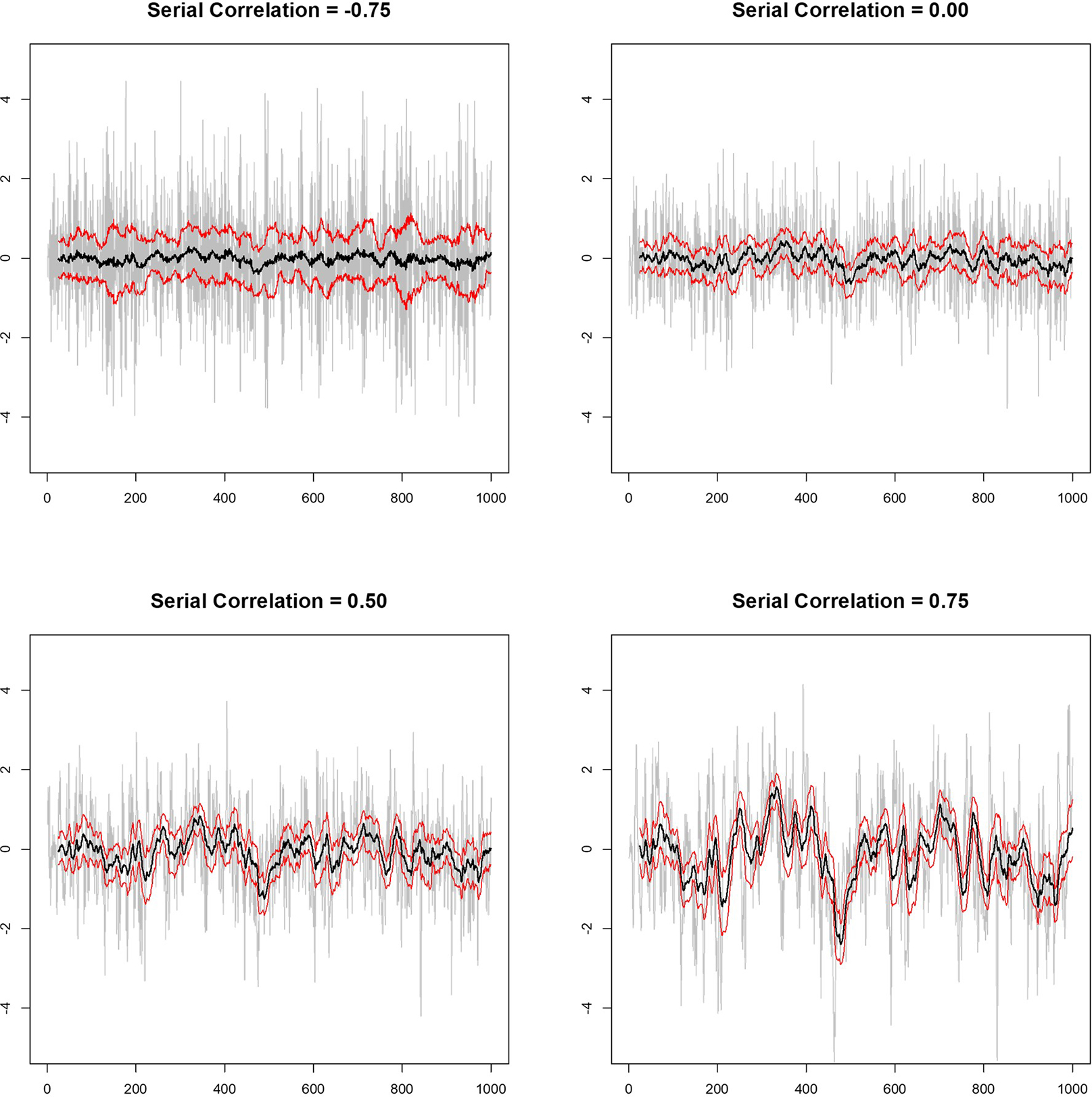
Four simulated random variates with varying degrees of AR-1 serial correlation. Time is on the *x*-axis and the value of the variate on the *y*-axis of each panel. The gray lines depict the variates, the black lines are the 25-measure rolling average of the variates over time, and the red lines give the upper and lower two times the standard error of the mean limits based on the 25-measure windows used to construct the rolling average.

Consider, Figure 1 (also compare with Figure 7.4 of (n.d.m)), which depicts the time series of a simulated random variate (in gray) generated under the assumption of different AR-1 serial correlation strengths using the R module ‘arima, sim’ (n.d.n). Note that R code for conducting these simulations and others described here are available upon request. Also depicted in Figure 1 are the 25-time-point rolling average of each series (as solid black lines) using the R module ‘roll,’ as well as twice the estimated standard error above and below the mean (as solid red lines) (n.d.o). It is clear from Figure 1 that the stronger the positive serial correlation, the more likely two randomly chosen segments will differ in their means in such a way that those means are not within the other segment’s local confidence limits about the mean, potentially leading to what would appear to be an intervention effect if certain analyses were pursued that did not account for the serial correlation. For example, consider the dip in the variate values with AR-1 serial correlation strength of 0.75 occurring between times ~200–225 and contrast that with the rise in the variate between times ~350–375. Variation in the local mean of the variable this pronounced is not observed in the variates with AR-1 serial correlation strengths of −0.75, 0.00 or even 0.50. If the 25 measures collected during times ~200–225 were obtained while an individual was provided intervention A and the measures collected during times ~350–375 were collected while that individual was provided intervention B, then one could falsely conclude that there is a difference in the measures that is attributable to the intervention if the serial correlation was not taken into account in the analysis of the data. We consider this phenomenon and its implications for personalized trials in more detail in the next section.

### 2.4. Simulation Studies Exploring Serial Correlation in Personalized Studies

In order to explore the effect of serial correlation on personalized studies, I simulated data in a couple of settings, starting with a more basic analysis setting and then considered more realistic analysis settings. Note that my simulation studies are in no way exhaustive or do justice to the myriad ways in which certain phenomena, like serial correlation, can impact the design and analysis of personalized trials. Rather, my goal is to expose the reader to various issues involving serial correlation effects. I assumed the basic N-of-1 design and linear regression analysis model with variance components described by (Rochon 1990) and (Wang and Schork 2019) to simulate the analysis of relevant personalized studies. However, in pointing out the untoward effects of serial correlation, I did not account for serial correlation in all the simulations and assumed the serial correlation was effectively 0.0 to determine the effect of this assumption on test statistics meant to capture an intervention effect. There are a number of features in personalized studies that could impact and be impacted by serial correlation if not accommodated for in any analysis (see Section 2.1). I consider a few of these features, including the use of washout periods and the length of washout periods, fixed vs. random order of the interventions, the number of crossover periods, and the number of measurements made in while the individual is on each of two interventions (A or B). The washout periods considered in the simulation studies were assumed to last as long as, or longer than, the intervention periods, but for which no response measurements were made so they were not included in the analysis. In this context, and in simulations in which the order of the treatments was randomized, a simulated intervention sequence could have constant, repeated alternations between treatments with washout periods, for example: ‘AWBWAWBWAWBWAWB’ where ‘W’ stands for a washout period, or could have a sequence with random alternations such as ‘AWAWBWAWBWBWBWA.’ The basic response measurement variable was assumed to follow a standard normal distribution with variance 1.0 and different degrees of serial correlation from 0.0 to 1.0. Random variates were generated using the R module ‘arima.sim’ as in Section 2.3.

#### 2.4.1. Basic Simulations

**Figure 2.**
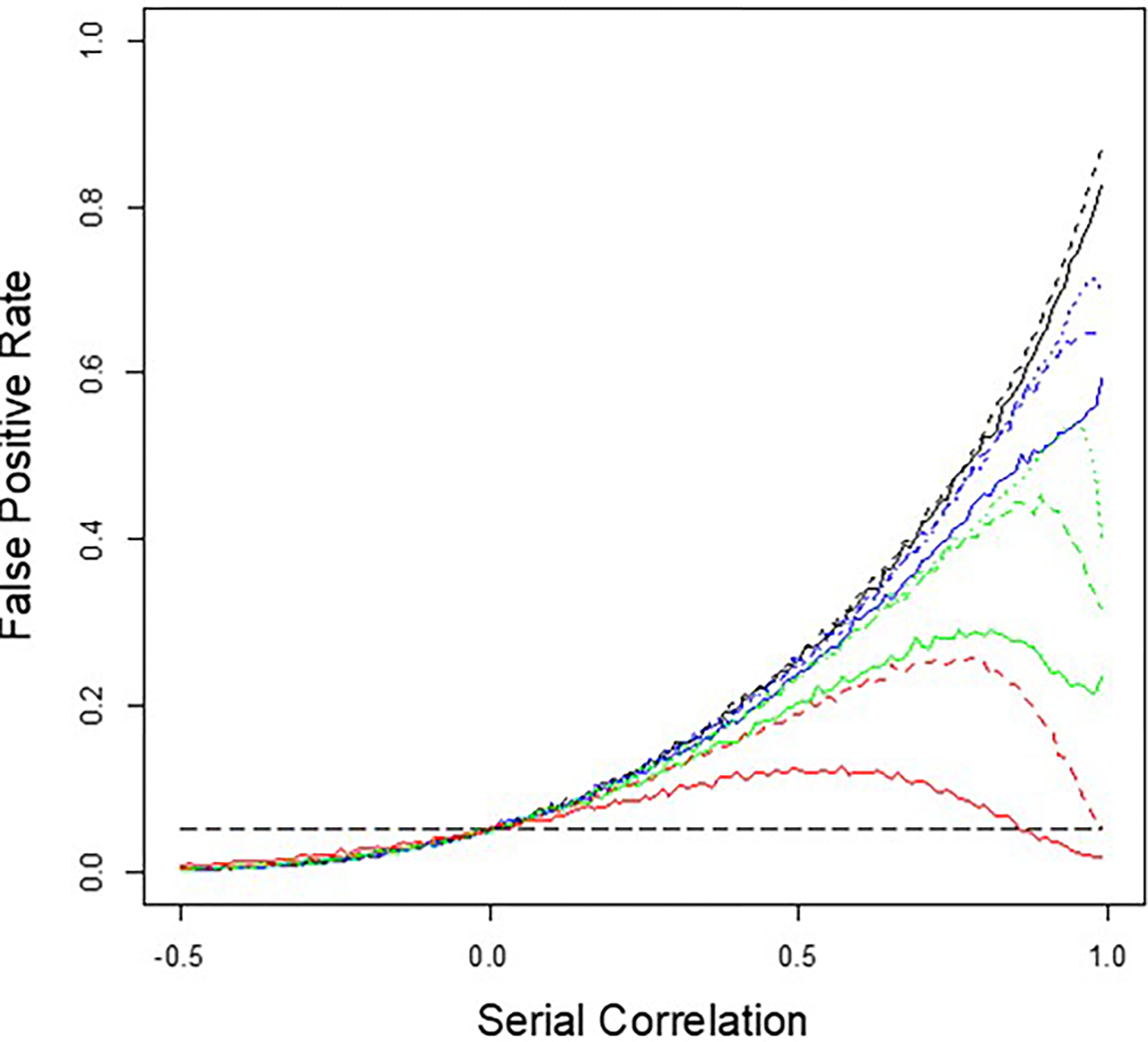
Graphical depiction of the simulation-based False Positive Rate of personalized studies with and without washout periods as a function of serial correlation strength between measurements. The long-dashed horizontal gray line is the assumed false positive rate of 0.05. The black lines represent the 2 × 1 × 40 designs with (dashed) and without (solid) washouts that take as much time as it does to collect 40 measurements; the blue lines represent the 2 × 2 × 20 designs with (dashed) and without (solid) washouts that are 20 measurements long and a setting with washouts 100 measurements long (dotted); the green lines represent the 2 × 4 × 10 designs with (dashed) and without (solid) washouts that are 10 measurements long and a setting with washouts of 50 measurements long (dotted); and the red lines represent the 2 × 4 × 5 designs with (dashed) and without (solid) washouts that are five measurements long.

I first simulated simple designs in which 40 measures were made while an individual was on each of the two interventions (80 measures total). I considered four different designs: 1. A design where the 40 measures for each intervention were made consecutively (a simple 2 intervention × 1 period × 40 measures design) with or without washouts between the interventions (AB vs. AWB) where the washout lasted a time equivalent to 40 measures; 2. A 2 × 2 × 20 design with and without washouts (ABAB vs. AWBWAWB); 3. A 2 × 4 × 10 design with and without washouts (ABABABAB vs. AWBWAWBWAWBWAWB); and 4. A 2 × 8 × 5 design with and without washouts (ABABABABABABABAB vs. AWBWAWBWAWBWAWBWAWBWAWBWAWBWAWB). I also simulated settings in which 2 × 2 × 20 and 2 × 4 × 10 designs were used with washout periods that lasted 100 measures and 50 measures, respectively. For each setting, I simulated series of 1,000 measures for serial correlation strengths between −0.99 and 0.99 increments of 0.01, randomly chose a point in each series as the start of a personalized trial, then assigned a dummy variable =0 to the 40 measures collected per intervention A and a dummy variable =1 to the 40 measures collected per intervention B. I then fit a simple linear regression model where the measures during intervention periods A and B were regressed on the 0/1 dummy variable. I did this 10,000 times for each assumed serial correlation strength and tallied the number of regression analyses in which the coefficient for the dummy variable was significantly different from 0.0 using a *t* test on the regression coefficient (based on the ‘lm’ module in R) at a type I of error level of 0.05. The results are depicted in Figure 2, which plots the fraction of times out of 10,000 that the tests of the dummy variable resulted in a *p*-value < .05 (the ‘False Positive Rate’ given that no real intervention effect was generated but rather one continuous series with serial correlation) against the serial correlation strength.

It can be seen from Figure 2 that the false positive rate of a regression-based test of an intervention that ignores serial correlation generally increases with increasing positive serial correlation, but decreases if the serial correlation is strongly negative. Note that the trend for the false positive rate goes to 0.0 for serial correlation strengths <−0.5 that approach 0.0, but the figure was purposely capped at −0.5 to highlight the influence of positive serial correlation. Note that for very large positive serial correlations, there is a dip in the false positive rate for designs in which the measurements for each intervention are collected in multiple shorter time segments (e.g., black lines = 2 × 1 × 40 measures vs. red lines = 2 × 8 × 5 measures). In addition, the use of washout periods exacerbates the false positive rate, as reflected in the solid lines (no washout times between intervention periods) vs. the dashed lines (washout periods) but in a manner that is dependent on the serial correlation strength.

There are some intuitive interpretations about the results of the simulation studies reflected in Figure 2: If a continuously monitored variable with zero mean and finite variance exhibits serial correlation, then there will likely be more ‘runs’ in which consecutive values of the variable are greater than, or lesser than, 0.0. This will be reflected in the mean during such a ‘stretch’ being greater than or lesser than 0.0 despite the fact that throughout the entire series (i.e., over all possible stretches within the series) the global mean is 0.0. Thus, randomly choosing two stretches of adjacent variable values separated by some number of measures (or time) could lead to differences in the mean of those numbers if attention is only confined to those stretches of adjacent values, certainly more so than when there is no serial correlation. When there is no interval between the two stretches of consecutive values of the variable (i.e., no washouts), this difference in the average segments has less of a chance of occurring, since any run of positive or negative numbers could bridge the two stretches or sequences of values and hence contribute to the average values during each of the two stretches. If the serial correlation is really pronounced, however, then any two stretches might still have values that are similar despite the washout period between them—note the dip in false positive rate for the green (2 × 2 × 20) and red (2 × 4 × 10) dashed line settings in Figure 2. However, if the washout periods are long relative to the times during which measures are collected during the intervention periods, then the values collected during those different intervention periods will not be as strongly correlated and hence likely differ by chance, increasing the false positive rate (as reflected in the blue and green dotted line settings).

#### 2.4.2. More Realistic Settings

In order to gain further insight into the effect of serial correlation on personalized trials and how it affects the power to detect a real effect of an intervention, as well as the false positive rate, of regression-based tests of an intervention effect, and how this effect might be remedied, I performed additional simulation studies. Here, I simulated 1,000 personalized N-of-1 trials with 400 total measurements assuming different correlation strengths as in section 2.4.1. Note that fewer simulation studies were done in each setting (1,000) relative to those pursued in section 2.4.1 (10,000) given the extra computational burden with the increased sample used and analytical methods used. As in section 2.4.1, a simulated intervention ‘A’ was compared to a comparator intervention ‘B’ with 50 response measures made during each of four intervention periods (i.e., 2 interventions × 4 periods × 50 measurements = 400 total observations). Studies for which the four periods for each intervention were randomized over the eight total intervention periods were also pursued, unlike the studies in section 2.4.1. An effect size of either 0.0 (no effect) or 0.3 (moderate effect) standard deviation units was assumed for intervention A relative to intervention B. Washout periods assuming a time equivalent to the collection of 50 measurement were also assumed in some simulations. Here, standard generalized least squares (GLS) regression analysis was used to relate measurements made during intervention period (coded as 0 for intervention A and 1 for intervention B) to the coded 0/1 intervention periods using the ‘gls’ module in the R package ‘nlme’ (n.d.p). Note that the gls model accommodates serial correlation by estimating it from the data along with regression coefficient parameters, although how it estimates this serial correlation when washout periods are used can raise questions, as we point out below. The power to detect an intervention effect assuming a type I error of 0.05 was determined by tallying the number of simulations out of the 1,000 resulting in a regression-based test of the intervention effect coefficient producing a *p*-value < .05. No carryover or other effects were simulated.

**Figure 3.**
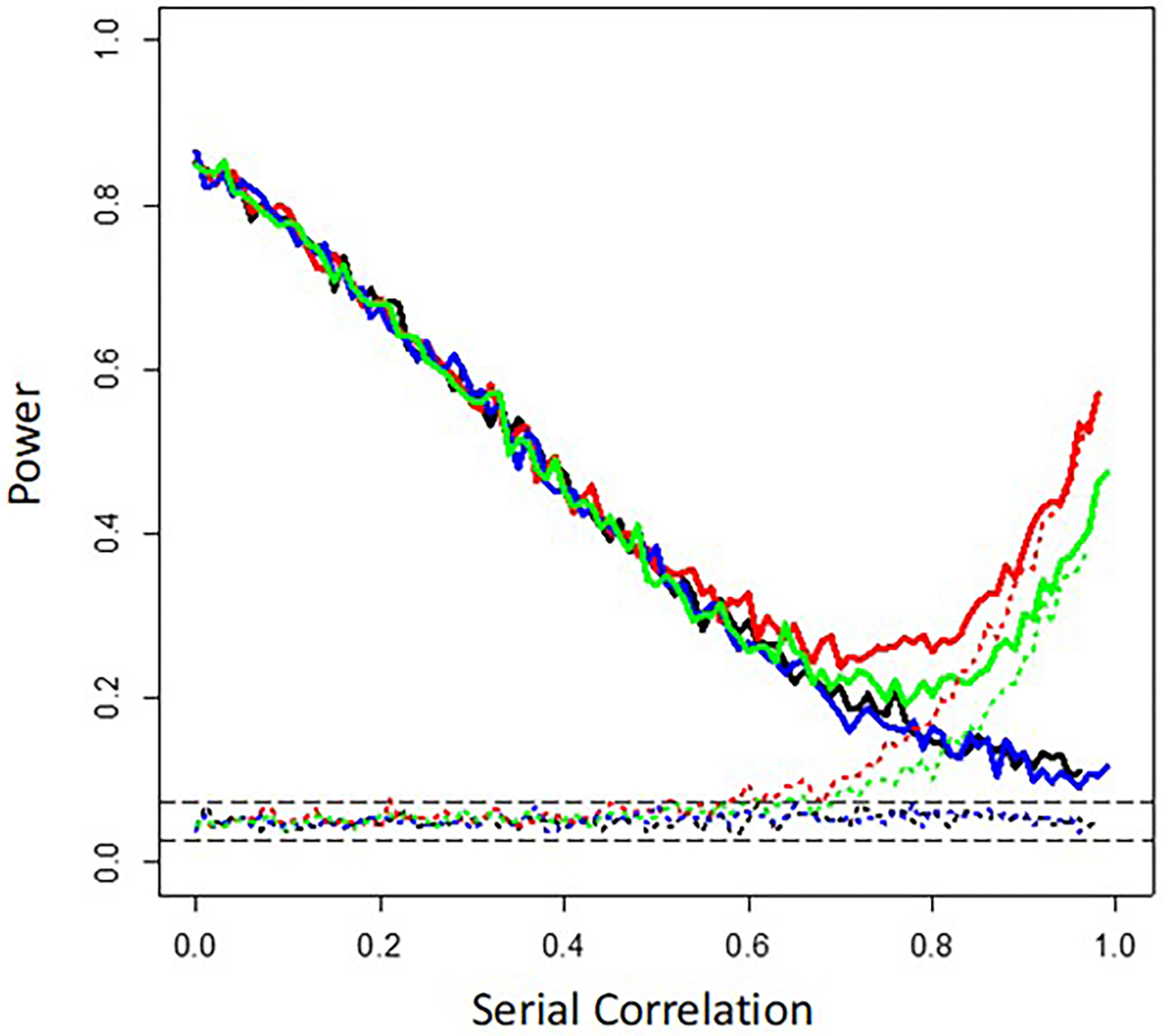
Graphical depiction of the results of simulation studies exploring the effect of serial correlation on N-of-1 studies with 400 total measurements for two interventions collected during four 50-measurement periods and without washout periods. The long-dashed horizontal gray lines reflect the theoretical 95% confidence bands surrounding an assumed false positive rate of 0.05. Irrespective of the color, the solid lines assume an effect size of 0.3 and the dotted lines an effect size of 0.0. The black lines assume no washout periods and no randomization of the order of the interventions; the blue lines assume no washout periods but intervention order randomization; the red lines assume washouts but no randomization; and the green lines assume both washouts and intervention order randomization.

The results are depicted in Figure 3. The different colored lines correspond to different assumptions about the use of washout periods and randomization of the order of the interventions, with the solid lines assuming an effect size of 0.3 and the dotted lines an effect size of 0.0. The black lines assume no washout periods and no randomization; the blue lines assume no washout periods but randomization; the red lines assume washouts but no randomization; and the green lines assume both washouts and randomization. The two long dashed gray lines provide theoretical 95% confidence limits for an estimate of a type I error rate of 0.05 given 1,000 simulations. It can be seen generally that the power of the gls regression coefficient-based test of an intervention effect is reduced when serial correlation increases. This is due to the fact that serial correlation essentially reduces the ‘effective’ number of measurements (or observations) because of their lack of independence, leading to a less powerful test relative to a test in which all the observations are independent (i.e., not serially correlated) (Bayley and Hammersley 1946)(Tang and Landes 2020)(n.d.q). For the settings involving washout periods and no assumed intervention effect (the dashed green and red lines in Figure 3), the ‘power’ (essentially the false positive rate) displays a sudden jump at a serial correlation strength of ~0.7 (contrast the red and green with the blue and black lines). This increase in false positive rates also manifests itself in the settings in which there is an assumed intervention effect (the solid red and green lines in Figure 3). Randomization mitigates this increase in false positives results (e.g., contrast the green and red lines). This jump in false positives when washouts are used likely arises because of the phenomena described in Section 2.4.1 and Figure 2, in which runs of correlated values of consecutive measurements are broken up, leading to random local differences in the average values in different time segments. This phenomenon raises questions about how to estimate serial correlation strength and account for it in personalized studies if the measurements are not collected in one continuous series but are rather broken up into smaller units, or, perhaps, if there are changes in serial correlation strength when interventions are rotated (e.g., some blood pressure medications may reduce variability in blood pressure over time, possibly affecting serial correlation between observations)—topics I do not consider further here.

#### 2.4.3. Accounting for Serial Correlation

**Figure 4.**
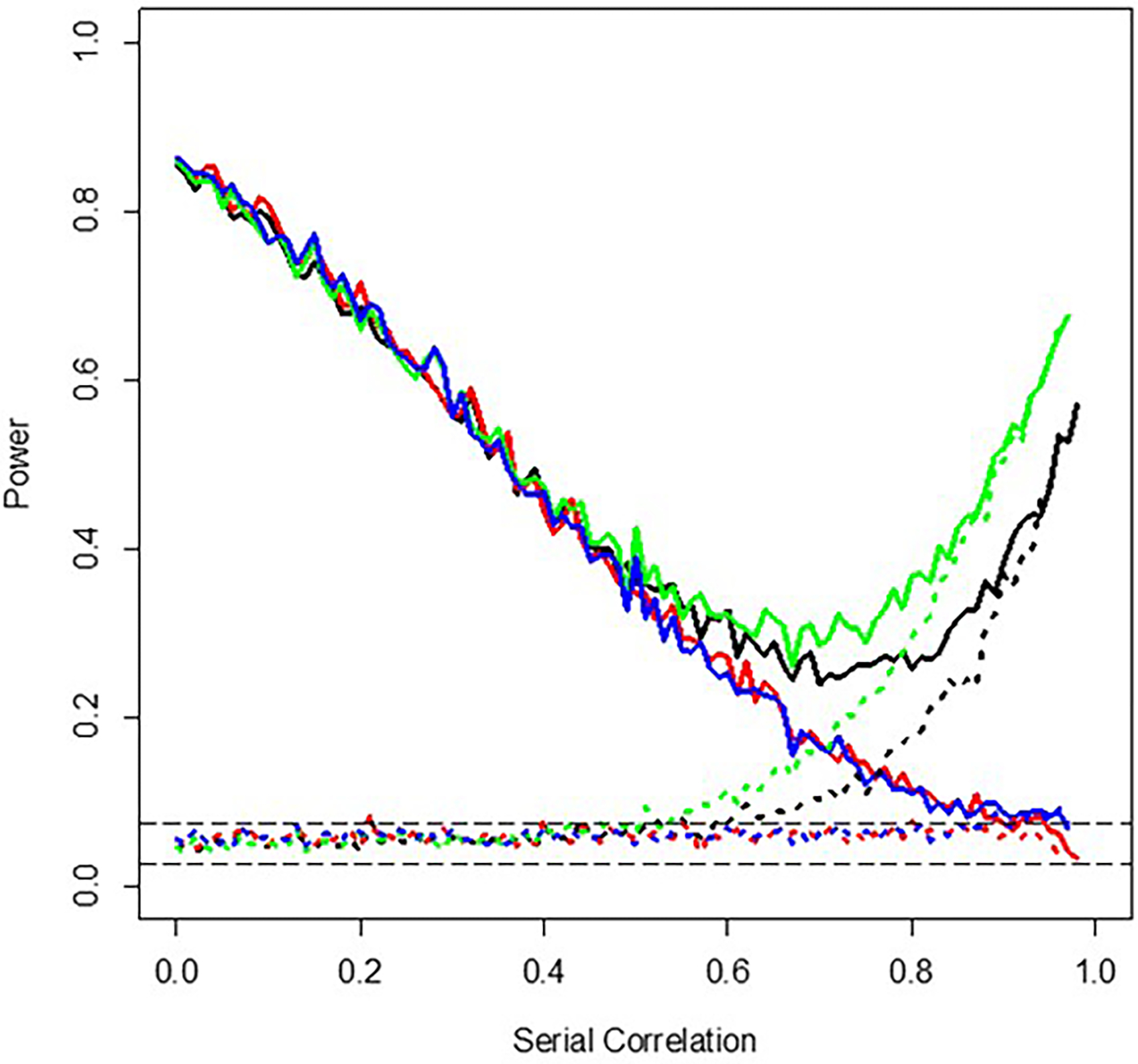
Graphical depiction of the results of simulation studies exploring the effect of serial correlation on Newey-West tests of intervention effects in personalized studies with 400 total measurements for two interventions collected during four 50-measurement periods and without washout periods. The long-dashed horizontal gray lines reflect the theoretical 95% confidence bands surrounding an assumed false positive rate of 0.05. The solid lines assume an effect size of 0.3 and the dotted lines an effect size of 0.0 with the black lines assuming washout periods and randomization using a standard, uncorrected for serial correlation, generalized least squares (GLS)-based test of the regression coefficient capturing the intervention effect; the green lines assume washout periods and no randomization using a standard GLS-based test; the red lines assume washouts and randomization but use of the Newey-West serial correlation-robust estimator of the intervention effect regression coefficient’s standard error in a test of the intervention effect, and the blue lines assume washouts but no randomization and use of the Newey-West estimator.

To explore how one can accommodate and control for serial correlation in the regression-based testing framework I have described, I considered tests of regression coefficients capturing intervention effects in linear models using the Newey-West serial correlation-robust estimator of regression coefficient standard errors (Whitney K. Newey and West 1987)(W. K. Newey and West 1994). The Newey-West test was carried out in these simulation studies using the module ‘coeftest’ in the R package ‘sandwich’ (Zeileis, Köll, and Graham 2020). Again, I simulated 1,000 settings in which a 2 × 4 × 50–measurement personalized trial was assumed with and without 50 measurement–long washout periods, for different assumed serial correlation strengths, and tallied the fraction of tests of regression coefficients associated with the intervention effect with *p*-values < .05. Figure 4 depicts the results of these simulations. Note that although the colors used in Figure 4 are also used in Figure 3, the lines are not necessarily capturing the same settings given that Figure 4 focuses on the use of the Newey-West–based tests, which are not considered in Figure 3. The different lines again correspond to different assumptions about the use of washout periods and randomization of the order of the interventions, with the solid lines assuming an effect size of 0.3 and the dotted lines an effect size of 0.0: the black lines assume washout periods and randomization using a standard, uncorrected for serial correlation, generalized least squares (GLS)-based test of the regression coefficient capturing the intervention effect; the green lines assume washout periods and no randomization using a standard GLS-based test; the red lines assume washouts and randomization but use of the Newey-West serial correlation-robust estimator of the intervention effect regression coefficient’s standard error in a test of the intervention effect and the blue lines assume washouts but no randomization and use of the Newey-West estimator. The use of the Newey-West estimator (red and blue lines) can clearly lead to more robust and appropriate inferences when serial correlation is present, although the power loss associated with reduced effective sample size due to serial correlation cannot be overcome. Greater attention to how to identify and accommodate serial correlation and its effects on personalized designs, especially those taking advantage of continuous monitoring devices, is needed.

## 3. Sequential N-of-1 Studies

### 3.1. Why Sequential Designs?

Many studies are designed with a fixed sample size determined prior to the initiation of the study based on available evidence about, for example, a possible effect size for the phenomenon of interest, potential confounding factors that need to be accommodated in various analyses, the statistical analysis model to be used, and so on. The use of a fixed sample size can be costly and inefficient if the phenomenon of interest—for example, the effect of an intervention on an individual in an personalized study—is more pronounced than thought and could have been detected with a smaller sample size. Alternatives to fixed sample size-based studies and test statistics involve sequential methods and tests, which evaluate a hypothesis (e.g., intervention effect vs. no intervention effect) after each measurement is made in real time. If the evidence for or against the hypothesis is overwhelming and statistically significant, the study is terminated. If there is not enough evidence to accept or reject a relevant hypothesis at any point, the sampling and measurements are continued. There are a number of approaches to sequential hypothesis testing (n.d.r)(Schnuerch and Erdfelder 2020)(n.d.s), but I focus on sequential probability ratio tests (SPRTs) here, which have been a mainstay in the field (Wald 1945)(Wald and Wolfowitz 1948). Most sequential analysis approaches, like SPRTs, posit boundaries informed by a priori specified fixed type I and type II error rates that, if crossed by the computed test statistic, will lead to termination of the study.

We note that there are modifications of SPRTs that are worth consideration from very practical perspectives. For example, to avoid continued sampling and measurement for very long periods of time if evidence for or against a hypothesis has not been obtained despite the accumulating data, one could modify the test to work with a maximum number of measurements which, if reached, would lead to the termination of the study while preserving the assumed type I and type II error rates for drawing inferences about the hypothesis of interest (Pramanik, Johnson, and Bhattacharya 2021). We also emphasize that if interest is *not* in making a decision about the efficacy of an intervention in the earliest time possible, but rather in exploring patterns and trends in a number of aggregated, independently pursued, personalized trials focusing on the same intervention or with the same overall design, there are many different methods for this (Blackston et al. 2019)(Punja et al. 2016), including those based on Bayesian meta-analyses in the present issue of *HDSR*.

In the following, I describe two examples of SPRTs in N-of-1 study contexts to showcase their potential. The first involves an evaluation of the population-level response rate to an intervention via sequentially aggregated N-of-1 study results with a limit imposed on the maximum number of personalized studies pursued over time. The second involves regression-based tests to detect an effect of an intervention in the shortest time possible. As with the results provided in the Section 2, I emphasize that they are not exhaustive, but rather meant to point out the potential for, for example, sequential analyses, and motivate further, more in-depth, studies.

### 3.2. Sequential Aggregated N-of-1 Studies for Overall Efficacy Claims

Consider a study in which interest is in determining both if an intervention benefits at least, for example, 20% of all individuals who it is provided to and, further, if there might be within-individual factors that influence responses to the intervention. Aggregated fixed–sample size personalized studies could be used for this purpose: one could simply tally the number of personalized studies, out of say 100 total that are pursued, in which the individuals studied exhibit a statistically significant response to the intervention and then see if this number is greater than or equal to 20%. Obviously, a definition of response would need to be provided, but that aside, such a study could also be pursued sequentially, where after each personalized trial, the count of how many individuals exhibited a significant response is tested to see if it is consistent with a 20% overall response rate. If it is, then the pursuit of the personalized trials is stopped. In the context of an SPRT, one could posit two hypotheses, H_1_: the response rate is, for example, 10% or less, and H_2_: the response rate is 20% or greater, and then determine if the evidence based on an SPRT test of the response rate at any point in time is: 1. consistent with H_1_ and the study should be stopped as a result; 2. consistent with H_2_ and the study should be stopped as a result; or 3. whether measurement and sampling should continue because there is not enough evidence in favor of either H_1_ or H_2_. We note that in such a study it is crucial to balance the type I and type II error rates posited for detecting the effect of the intervention on an individual studied in a personalized study with the type I and type II error rates posited for determining if the response rate across the personalized studies is consistent with the overall rates of response, H_1_ or H_2_.

I explored some of the properties and behavior of a modified SPRT to evaluate a response rate to an intervention via sequentially aggregated personalized studies. The modification assumed a maximum number of personalized studies used to evaluate, estimate, and test the overall response rate, which we set at 100 (Pramanik, Johnson, and Bhattacharya 2021). I simulated personalized trials involving 400 total measurements with four intervention periods for two interventions, as considered in Sections 2.4.2–2.4.4. I did not consider the use of washout periods, randomization of the order in which the interventions were provided, carryover effects, or serial correlation, but these phenomena should clearly be explored. I assumed that some fraction of individuals participating in each trial would exhibit an effect of intervention A, which would amount to a 0.3 standard deviation–unit increase in the response measure relative to intervention B, as in Sections 2.4.2–2.4.4. The test of the hypothesis that intervention A would induce an effect was performed with a standard generalized least squares regression test, as also considered in Section 2. Either a type I error rate of 0.001 or 0.05 was assumed for these tests. Hypotheses were posited for the overall response rate, H_1_: response rate = 0.01 vs. H_2_: response rate = 0.05. We also considered H_1_: response rate = 0.05 vs. H_2_: response rate = 0.1 as well as H_1_: response rate = 0.1 vs H_2_: response rate = 0.2. The simulations assumed that the actual response rate was between 0 and 1.0 in steps of 0.01. Only 100 simulations for each setting were pursued due to the computational burden and the number of times H_2_ was accepted was taken as an estimate of the power of the modified SPRT of the response rate. Assumed overall error rates for the modified SPRT of the response rate were set to 0.05.

**Figure 5.**
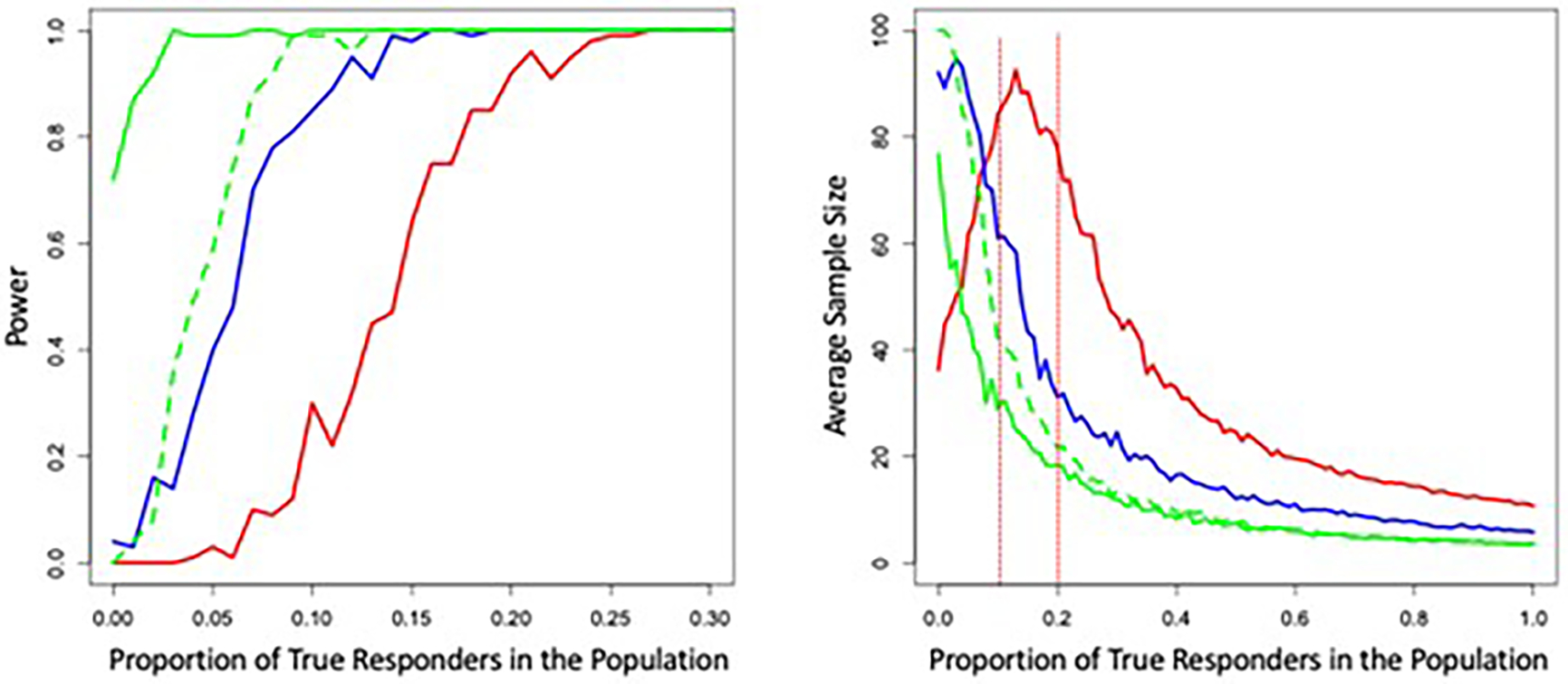
Graphical depiction of the results of simulation studies exploring the power of sequential tests of intervention response rates obtained from consecutive personalized studies as a function of the true response rate. The solid green line assumed H_1_: response rate = 0.01 and H_2_: response rate = 0.05 and a type I error rate for a test of an individual’s response to an intervention in a single personalized study of 0.05. The dashed green line assumed H_1_: 0.01 and H_2_: 0.05 and a type I error rate for a of 0.001. The solid blue line assumed H_1_: 0.05 and H_2_: 0.1 and a type I error rate of 0.05. The red line assumed H_1_: 0.1 and H_2_: 0.2 and a type I error rate of 0.05. The right panel of Figure 2 depicts the average sample sizes (i.e., total number of personalized trials pursued sequentially) for each setting depicted in the left panel of Figure 5, with the same color coding.

The left panel of Figure 5 depicts the relationship between simulation-based estimates of the power of the modified SPRT to accept H_2_ as a function of the true proportion of responders. The solid green line assumed H_1_: response rate = 0.01 and H_2_: response rate = 0.05 and a type I error rate for a test of an individual’s response to an intervention in a single personalized study of 0.05. The dashed green line assumed H_1_: response rate = 0.01 and H_2_: response rate = 0.05 and a type I error rate for a test of an individual’s response to an intervention in a single personalized study of 0.001. The solid blue line assumed H_1_: response rate = 0.05 and H_2_: response rate = 0.1 and a type I error rate for a test of an individual’s response to an intervention in a single personalized study of 0.05. The red line assumed H_1_: response rate = 0.1 and H_2_: response rate = 0.2 and a type I error rate for a test of an individual’s response to an intervention in a single personalized study of 0.05. The left panel of Figure 5 clearly shows that the power to detect a response rate consistent with H_2_ increases with the true response rate, as expected. In addition, comparison of the solid and dashed green lines suggests that if the type I error rate for detecting an intervention response is not well below the assumed H_1_ and H_2_ response rates, then an increase in false positive H_2_ acceptances will occur, again as expected. The right panel of Figure 5 depicts the average sample sizes (i.e., total number of personalized trials pursued sequentially) for each setting depicted in the left panel of Figure 2, with the same color coding. It is clear from the right panel of Figure 5 that a substantial savings, in terms of the number of personalized studies that need to be pursued, can result from sequential testing of the response rate. The red vertical lines indicate the H_1_ and H_2_ response rates for the setting with them set at 0.1 and 0.2 and suggests that the largest required sample sizes occur when the actual response rate is intermediate between H_1_ and H_2_ which is intuitive, since this rate is hardest to distinguish between the two hypotheses and is associated with a power of roughly 0.5, which reflects a balance between accepting H_1_ and accepting H_2_.

## 3.3. Quickest Single-Subject Outcome Determination Studies

Consider studies in which one wants to determine if there is any evidence of an effect of a single intervention on an individual in the shortest amount of time possible. This setting departs from traditional personalized or traditional N-of-1 studies in that it may not involve a comparator intervention. However, this setting is appropriate when there is urgency in decision-making or it is problematic to cross-over an individual to a different intervention merely for statistical expediency. As an example, consider treating an individual cancer patient and wanting to know if an intervention is actually shrinking that patient’s tumor. In this setting, knowing that an intervention is not working in the shortest amount of time possible is crucial for the patient’s life and testing a new intervention simply for statistical considerations would likely be unethical if the initial intervention looks as though it is working. As another example, consider testing food-based cognitive enhancer (Onaolapo, Obelawo, and Onaolapo 2019) on cognitive abilities, where every so often an individual takes, for example, a reaction time test, after consuming the cognitive enhancer of interest. In this situation, the likely implementation of the study is fairly simple and not ethically complicated, and yet a participant and researcher might want to know if something positive is occurring in a relatively short period of time so as to not waste time with the study. If, after a certain period of time, there is ample evidence of, for example, tumor shrinkage or cognitive enhancement, one could infer that the intervention has an effect and terminate the study at that point and ultimately save costs associated with the continued measurement of tumor size or evaluating reaction time. Obviously, one would need to be sensitive to covariate effects and other likely confounders in interpreting the results of such a study, however.

Developing an appropriate test statistic to detect an effect of an intervention soon after its administration is not entirely trivial, but can be framed as a regression problem. If the belief is that the intervention will affect an outcome measure like blood pressure, weight, tumor size based on imaging protocols, mood, sleep quality, or reaction time to a greater degree as time goes on since the administration of the intervention until a point of maximal effect is reached, then one would expect to see a relationship between time since the administration of the intervention and the response measure. This relationship may be negative (e.g., if the intervention was designed to lower blood pressure or weight) and could be tested for its statistical significance via regression methods by determining if the slope of the regression of the outcome measure on time since initiation of the intervention is greater than or equal to a specific value. Note that in the setting, time or ‘learning’ effects of the type briefly mentioned Section 2.1 could confound valid inference and should be acknowledged and minimized if possible. Pursuing tests of slopes in regression models sequentially via SPRTs is complicated by the fact that fitting a regression model to estimate a single slope requires estimation of a *y*-intercept term, any covariate regression coefficients, and/or residual error terms. Appropriate implementation of SPRTs requires an understanding of the behavior of the SPRT statistic so its variance under null conditions can be used to inform the creation of thresholds which, if the SPRT statistic crosses, can in turn be used to determine if H_1_: slope for the response variable/time relationship = 0.0 vs. H_2_: slope > some value of interest should be accepted. Naively using an SPRT that does not consider the noise in the behavior of the SPRT statistic attributed to the estimation of the nontarget-slope ‘nuisance parameters’ would lead to tests resulting in false positive and false negative findings (n.d.t)(Gölz, Fauss, and Zoubir 2017).

**Figure 6.**
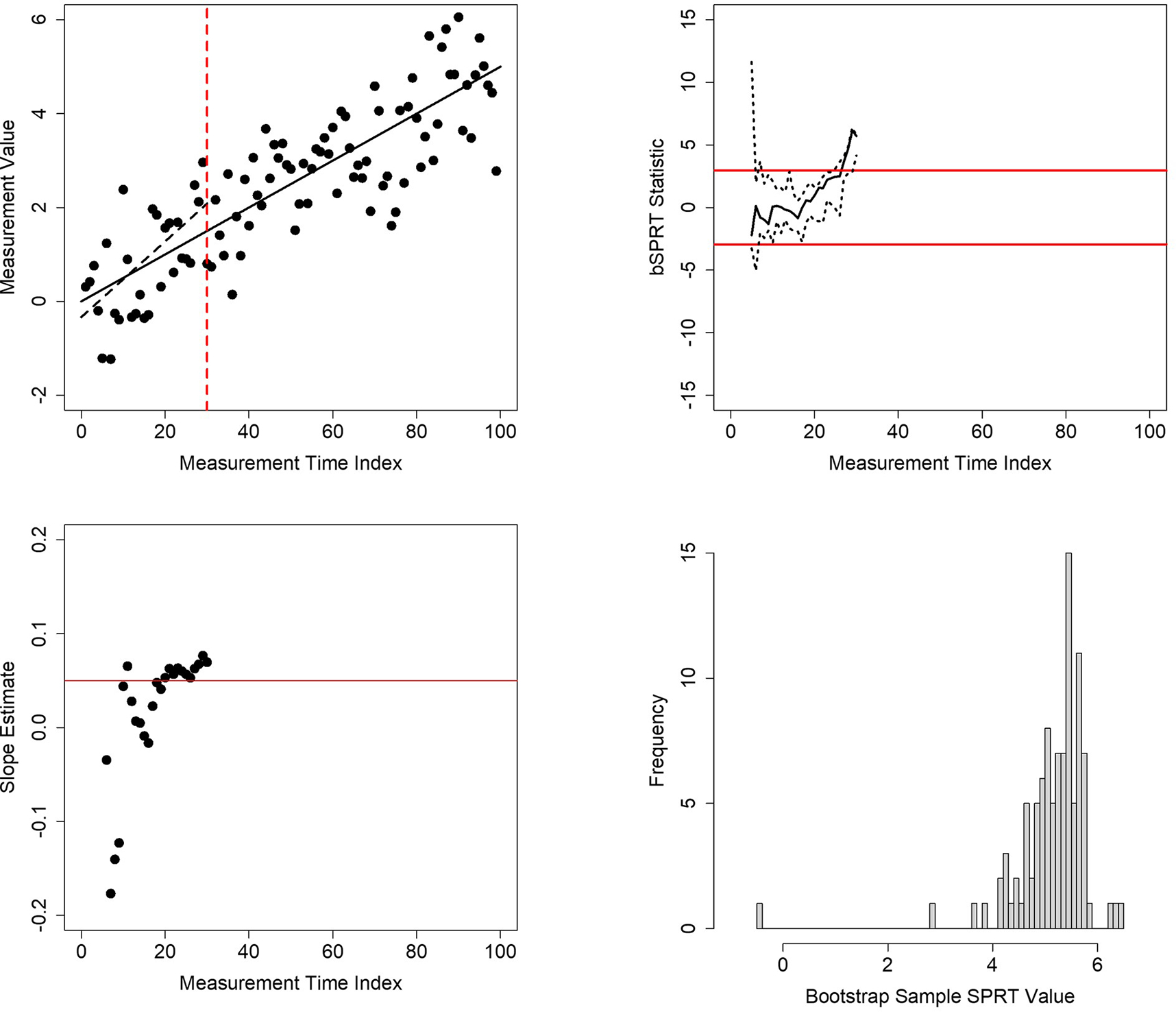
Results of a single simulation of a bootstrapped-based sequential probability ratio test (bSPRT) of a regression coefficient. Upper left panel: 100 simulated measurement values plotted against time of collection. The solid black line is the known regression relationship between the values and time. The dashed black line is the estimated regression relationship for the first 31 observations. The vertical red dashed line is the time at which evidence for the slope of the regression between the values and time reached statistical significance (i.e., time 31). Upper right panel: the behavior of the SPRT statistic evaluating the hypothesis that the slope computed from a regression analysis of the numbers in the upper left panel is not equal to zero (black line) and its 95% confidence limits (dashed black lines). The red lines give the crossing boundaries for significance at a type I error rate of 0.05. Lower left panel: estimate of the slope after each successive measurement until time 31. Lower right panel: distribution of the SPRT statistic based on 100 bootstrap samples at time 31.

Working out the thresholds used to determine if H_1_ or H_2_ should be accepted analytically can be complicated (Gölz, Fauss, and Zoubir 2017). However, bootstrap methods can be used to characterize the behavior of the SPRT statistic and create approximate thresholds for an SPRT, including an SPRT for testing a single regression coefficient as considered here. This strategy simply estimates confidence limits of the SPRT statistic using bootstrap sampling at each time a measurement is made in the study (Gölz, Fauss, and Zoubir 2017). Basically, after an evaluation of the SPRT statistic under an assumed pair of H_1_ and H_2_ values for the slope (e.g., H_1_: slope=0.0 and H_2_: slope=0.05), bootstrap samples are drawn with replacement from the current accumulated set of measurements and measurement times. The SPRT is calculated with these bootstrapped samples using the estimated regression coefficient parameters evaluated under H_1_ and H_2_ obtained from the actual data; that is, *not* those reestimated with the bootstrap samples. Fixed upper and lower percentiles of the estimated SPRT statistic distribution from the bootstrapped SPRT statistics are then obtained (e.g., the 5th and 95th percentiles) depending on desired type I and type II error rates for the SPRT. If the threshold for accepting H_1_ (positing a value lower than H_2_), as determined in a standard nonbootstrapped SPRT (n.d.u)(Gölz, Fauss, and Zoubir 2017), is crossed by the *upper* confidence limit of the estimated SPRT distribution, or if the threshold for accepting H_2_ (positing a value higher than H_1_) is crossed by the *lower* confidence limit of the estimated SPRT distribution, the SPRT is terminated. The upper right panel of Figure 6 depicts this phenomenon. Use of these bootstrap confidence limits instead of the actual SPRT statistic should preserve the assumed type I and type II error rates of the SPRT (Gölz, Fauss, and Zoubir 2017). The strategy of using the parameter estimates obtained with the actual data to compute relevant likelihood or probability ratio test statistics from bootstrap samples in order to evaluate the distribution of that test statistic is not necessarily the norm, but has been used in other contexts; for example, I used it some time ago (~33 years ago) in the development of bootstrap-based tests of separate families of hypotheses in genetic analysis settings (n.d.v).

To explore the properties of a bootstrapped-based SPRT (referred to here as the ‘bSPRT’) of a regression coefficient for personalized studies to determine the effect of an intervention in the shortest time possible, I conducted a few simulation studies. Figure 6 provides an example of a single simulated bSPRT. The upper left panel of Figure 6 provides a scatterplot of the simulated outcome variable against the time since the initiation of the intervention. It was assumed that the slope of the regression of the outcome on time was 0.05. The solid black line is the true regression line, with slope of 0.05 and *y*-intercept 0.0. The residual values of the regression of outcome on time were assumed to follow a standard normal distribution. The red dashed vertical line gives the time (measurement time 31) at which the bSPRT terminated when evaluating the slope under H_1_: slope=0.01 and H_2_: slope=0.05. The dashed black line is the regression line estimated from the data at the time 31. The upper right panel provides the bSPRT statistic applied to the data reflected in the upper left panel computed with the data available at each timepoint as well as its estimated upper 95% and lower 5% confidence intervals from 100 bootstrap samples. The red lines given the thresholds for accepting H_1_ (lower threshold) and H_2_ (upper threshold). It can be seen from the upper left panel of Figure 6 that although the nonbootstrapped SPRT statistic crossed the upper threshold at around time 28 or 29, the lower confidence interval of the bSPRT estimated from the bootstrap samples did not cross this threshold until time 31. The lower left panel provides the estimated slopes of the regression of the outcome variable on time at each time point and shows that the slope estimates were approaching, if not surpassing, the H_2_: slope=0.05 value over time. The lower right panel provides a histogram of the bSPRT statistics obtained from the bootstrap samples at time 31.

I explored the power and type I error rates of a bSPRT for a regression coefficient as well through simulation studies. I simulated 100 bSPRTs using 100 bootstrap samples to estimate upper (95%) and lower (5%) confidence limits of the SPRT at each time point for each of the simulated bSPRTs. I did this assuming the effect size of the intervention (i.e., the slope of the regression of outcome on time since initiation of the intervention) was 0.0 (the null case), 0.01, 0.025, 0.04, 0.05, and 0.075. I also assumed serial correlation levels of 0.0 and 0.5. Table 1 provides the results of these simulation studies and includes the assumed effects sizes (‘Effect Size’), serial correlation (‘Serial Correlation), average number of measurements at the time of termination of the bSPRT (‘Average SS), the standard deviation of the number of measurements at the time of termination of the bSPRT (SD SS), the number of simulated SPRTs for which H_1_ was accepted (‘Accept H_1_’) and H_2_ (‘Accept H_2_’) for both an SPRT that did not use bootstrapped estimated confidence limits (‘Standard SPRT’) and that did (‘Bootstrap SPRT’). I also included the average and standard deviation of the estimates of the serial correlation at the time of the termination of the SPRTs over the simulations (‘AveSerCor’ and ‘SDSerCor’). It can be seen from Table 1 that the bSPRT has better control over false positive rates (i.e., the number of times H_1_ was accepted when the true slope was 0.0) than the standard nonnuisance parameter-corrected SPRT in that its rate is closer to the assumed type I error rate value 0.05. In addition, the bSPRT has better power than the standard SPRT, due to early and erroneous terminations of the standard SPRT attributable to noise in the standard SPRT statistic due to estimating nuisance parameters. Serial correlation does have an effect, but it is much less pronounced for the bSPRT. The results of the simulation studies suggest potential for the bSPRT in the context described, but clearly more simulations involving more bootstrap samples to estimate SPRT statistic confidence limits, a broader range of effect sizes and serial correlation levels, as well as the use of tests of a regression coefficient that might be robust to serial correlation, are in order.

## 4. Future Directions in N-of-1 Studies

There is an excellent case to be made that if personalized (i.e., individualized and/or precision) medicine is to advance—whether focusing on individuals with rare diseases or the health improvement of individuals with all sorts of health concerns in the real world through tailored dietary, activity, and stress reduction interventions—personalized or N-of-1 studies will have a prominent role to play. However, there is a need to address some important and potentially confounding phenomena in such trials, such as serial correlation among the response observations (Section 2), as well as the efficiency and cost-effectiveness of such studies (Section 3). More reliable and efficient personalized trials will be needed to assess the utility of many emerging interventions such as those mentioned in Section 3.2 that require tailoring of the intervention to an individual’s possibly unique profile, such as antisense oligonucleotide (ASO)-based therapies, CAR-T cell therapies, gene therapies, and general personalized health optimization strategies (Nicholas J. Schork et al. 2020). The use of personalized interventions will also force the community to consider if the effort for achieving personalization is worth it in any given context (e.g., treating diabetes, cancer, depression). This question about the degree to which an intervention that requires personalization works could be addressed by pursuing a series of aggregated personalized studies and then pursuing a meta- or mega-analysis of the data and results of the aggregated studies. In this way, the number of individuals that benefit from the tailored approach could be assessed, and this could possibly be pursued sequentially, as described in Section 3.2. In addition, given the growing availability of wireless health monitoring devices and internet-of-things (IoT)–based infrastructure for collecting health status information (Cha et al. 2019)(Chung, Fortunato, and Radacsi 2019)(Marra et al. 2020), one could envision very large initiatives in which personalized studies are pursued remotely on individuals (e.g., evaluating the effects of cognitive enhancers on cognitive decline using apps and internet-based cognitive tests as discussed in Section 3.3), with no face-to-face contact of an enrollee in the trial with the team conducting the studies. One could further imagine that the results of those trials, as they are completed, being monitored in real-time with online false discovery rate (FDR) strategies to ensure that inferences drawn from them about, for example, population-level benefits of the interventions, are robust and not resulting in false positive and negative claims (Robertson et al. 2019). It may even be possible to pursue each individual personalized study meant to be aggregated with others in a sequential manner, so that the time involved in the study for any one individual is also minimized, as discussed in Section 3.3.

## Figures and Tables

**Table 1.** Results of simulation studies of a bootstrapped-based sequential probability ratio test (bSPRT) for a regression coefficient.

		Standard SPRT				Bootstrap SPRT					

Effect Size	Serial Correlation	Average SS	SD SS	Accept H_1_	Accept H_2_	Average SS	SD SS	Accept H_1_	Accept H_2_	AveSerCor	SD SerCor
0.000	0.000	24.800	11.081	0.830	0.170	35.710	8.262	0.990	0.010	0.052	0.073
0.010	0.000	25.480	12.634	0.757	0.2433	40.707	11.152	0.927	0.073	0.046	0.067
0.025	0.000	31.460	16.937	0.570	0.430	50.930	14.377	0.490	0.510	0.037	0.047
0.040	0.000	26.270	12.761	0.210	0.790	40.040	10.357	0.070	0.930	0.042	0.056
0.050	0.000	23.110	11.460	0.090	0.910	34.930	9.160	0.000	1.000	0.048	0.078
0.075	0.000	18.810	7.507	0.070	0.930	28.380	5.674	0.000	1.000	0.038	0.059
0.000	0.500	14.138	9.074	0.710	0.290	33.508	13.398	0.898	0.103	0.299	0.191
0.010	0.500	14.100	9.952	0.550	0.450	38.700	17.072	0.780	0.220	0.311	0.163
0.025	0.500	14.630	10.020	0.520	0.480	43.830	21.811	0.530	0.470	0.339	0.195
0.040	0.500	15.820	11.780	0.340	0.660	39.870	16.019	0.250	0.750	0.311	0.181
0.050	0.500	14.700	9.375	0.310	0.690	35.150	12.726	0.130	0.870	0.301	0.186
0.075	0.500	13.060	8.803	0.250	0.750	31.780	11.842	0.030	0.970	0.304	0.198

*Note.* ‘Effect Size’ is the assumed effect size; ‘Serial Correlation’ is the assumed serial correlation; ‘Average SS’ is average number of measurements at the time of termination of the bSPRT; ‘SD SS’ is standard deviation of the number of measurements at the time of termination of the bSPRT; ‘Accept H1’ is the fraction of simulated SPRTs for which H1 was accepted; ‘Accept H2’ is the fraction of simulated SPRTs for which H2 was accepted. ‘Standard SPRT’s did not use bootstrapped estimated confidence limits and ‘Bootstrap SPRT’s did. ‘AveSerCor’ and ‘SDSerCor’ are the average and standard deviation of the estimates of the serial correlation at the time of the termination of the SPRTs over the simulations.
